# Gradual ulnar lengthening in Masada type I/IIb deformity in patients with hereditary multiple osteochondromas: a retrospective study with a mean follow-up of 4.2 years

**DOI:** 10.1186/s13018-020-02137-z

**Published:** 2020-12-09

**Authors:** Yuchan Li, Zhigang Wang, Mu Chen, Haoqi Cai

**Affiliations:** Department of Pediatric Orthopedics, Shanghai Children’s Medical Center, Shanghai Jiao Tong University School of Medicine, 1678 Dongfang Road, Shanghai, 200127 People’s Republic of China

**Keywords:** Gradual ulnar lengthening, Forearm deformity, Radial head dislocation, Recurrence, Hereditary multiple osteochondromas

## Abstract

**Background:**

Gradual ulnar lengthening is the most commonly used procedure in the treatment of Masada type I/II deformity in patients with hereditary multiple osteochondromas. However, the treatment remains controversial for the recurrence of deformity in growing children. This study aims to evaluate the clinical and radiological outcomes of ulnar gradual lengthening in our clinic.

**Methods:**

We retrospectively reviewed patients who underwent ulnar lengthening by distraction osteogenesis from June 2008 to October 2017. The carrying angle (CA) and range of motion (ROM) of the forearm and elbow were clinically assessed, and the radial articular angle (RAA) and ulnar shortening (US) were radiologically assessed before lengthening, 2 months after external frame removal, and at the last follow-up.

**Results:**

The current study included 15 patients (17 forearms) with a mean age of 9.4 ± 2.3 years at the index surgery. The mean follow-up period was 4.2 ± 2.4 years. There were 9 patients (10 forearms) with Masada type I deformity and 6 patients (7 forearms) with Masada type IIb deformity. The mean amount of ulnar lengthening was 4.2 ± 1.2 cm. The mean RAA improved from 37 ± 8 to 30 ± 7° initially (*p* = 0.005) and relapsed to 34 ± 8° at the last follow-up (*p* = 0.255). There was a minimal deterioration of US yet significant improvement at the last follow-up compared to pre-op (*p* < 0.001). At the last follow-up, the mean forearm pronation and elbow flexion increased significantly (*p* < 0.001 and *p* = 0.013, respectively), and the mean carrying angle also improved significantly (*p* < 0.001). No patient with type IIb deformity achieved a concentric radial head reduction.

**Conclusions:**

Gradual ulnar lengthening significantly reduces cosmetic deformity and improves function in patients with Masada type I/IIb deformity. Our results supported early ulnar lengthening for patients with a tendency of dislocation of the radial head.

## Background

Hereditary multiple osteochondromas (HMO) is an autosomal dominant condition characterized by multiple benign cartilage-capped tumors, which typically occur at the juxta-epiphyseal region of the tubular bones. Forearm deformities resulting from tumor-induced growth disturbances of the proximal and distal radial and the distal portion of the ulna are common, with a prevalence of 40–74% of HMO patients [1]. Masada et al. classified forearm deformities into three types [2]. In type I, the main osteochondroma is located in the distal ulna and results in ulnar shortening and ulnar deviation at the wrist, with a secondary bowing radius often observed. Type II is ulnar shortening with a dislocated radial head; this type can be further divided into two subgroups: type IIa (proximal radial osteochondroma involved) and type IIb (no proximal radial osteochondroma involved). In type III, the main osteochondroma involves the distal part of the radius with a relative shortening of the radius. Because of the cross-sectional diameter of the distal ulnar physis being smaller than the radius, the ulna is more vulnerable to growth impairment [2], and therefore, type I and type II are more common.

A shorter proportional ulnar length is associated with a diminished range of motion of the forearm in type I, while in type II, the deformities result in the restriction of both elbow movement and forearm rotation [1, 2]. Gradual ulnar lengthening has been widely used with successful reported results in managing forearm deformity [3–7]. However, the treatment remains controversial. The current study aims to evaluate our mid-term clinical and radiological outcomes of gradual ulnar lengthening for Masada type I/II deformity, and we hypothesized that simple gradual ulnar lengthening would effectively improve the cosmetic problems and forearm function for Masada type I/II deformity.

## Methods

**Fig. 1:**
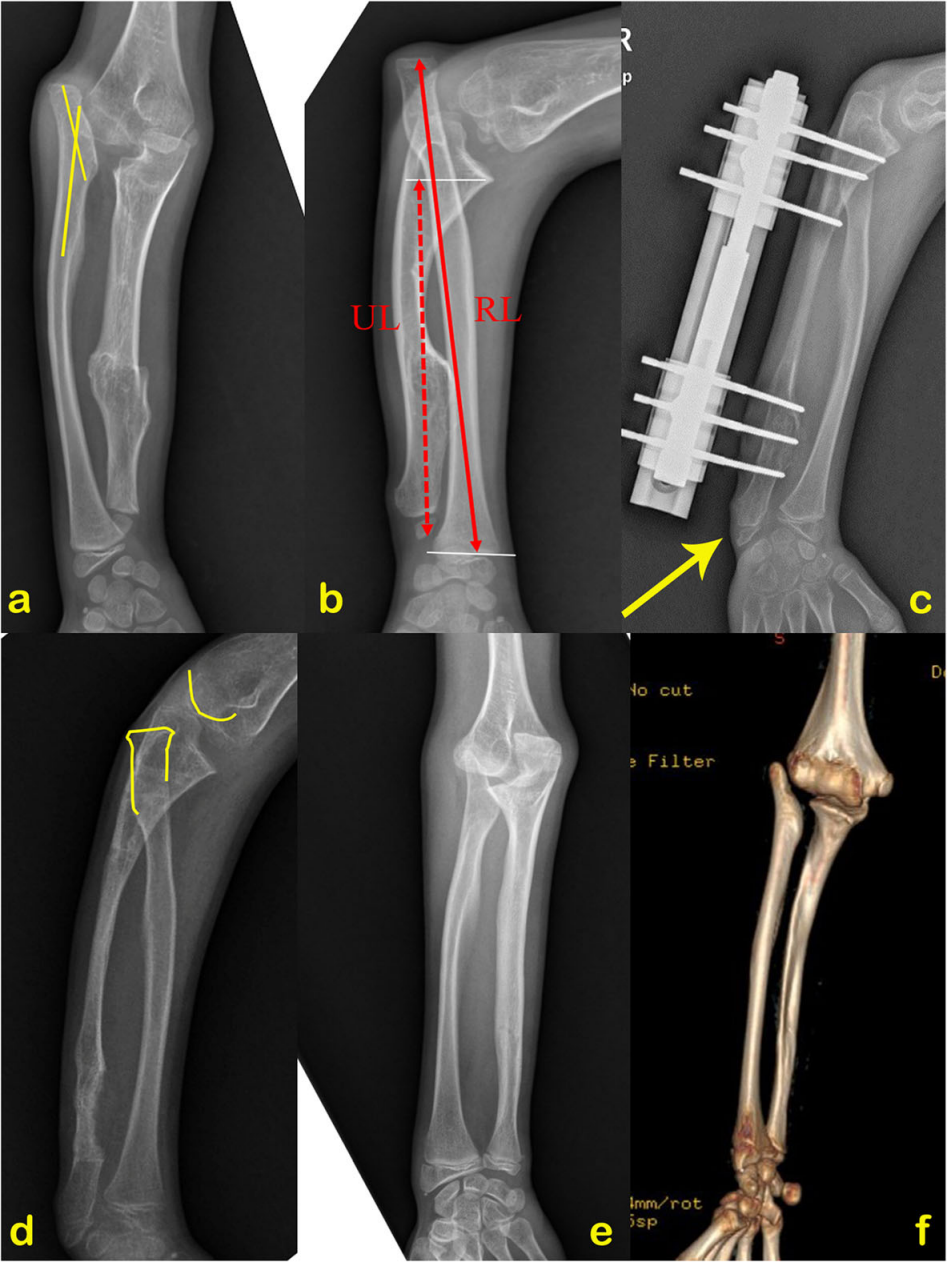
**a** Type IIb deformity in a patient with HMO, adaptive lateral angulation of the proximal radius due to the long-standing dislocation of the radial head. **b** In the lateral view, radial length (RL) is the distance from the center of the proximal radial physis to the center of the distal radial physis. Ulnar length (UL) is the distance between the trochlear and the ulnar styloid on the longitude axis of the ulna; ulnar shortening was determined by the RL minus the UL. **c**, **d** The ulna was overlengthened for 15 mm, and the radial head was not reduced by gradual ulnar distraction. **e**, **f** The patient came back 3.1 years after surgery, and computed tomography (CT) was performed, demonstrating the posterolateral displacement of the radial head, but the distal radioulnar joint was remolding well

This study received institutional review board approval. We retrospectively reviewed the medical records of all patients with HMO who underwent gradual ulnar lengthening in our hospital from June 2008 to October 2017. Patients with full radiographs (the anteroposterior and the lateral radiographs) pre- and postoperatively were included in this study, and patients with less than 2 years of follow-up were excluded. The forearm deformity was classified according to Masada et al.’s classification [2]. Demographic data and chief complaints of patients prior to the operation were recorded. Clinical results included the carrying angle (CA) and range of motion (ROM) of the forearm and elbow, compared between preoperative and the last follow-up. The radiological assessment included the radial articular angle (RAA) [8], as measured on the anteroposterior (AP) radiograph, and ulnar shortening (US), as measured on the lateral radiograph (Fig. 1); these data were recorded before lengthening, 2 months after the external frame removal, and at the last follow-up. The concentric reduction was defined as a line drawn through the center of the radial neck and should extend directly through the center of the capitellum both on the AP and lateral radiographs.

The operative procedure included gradual ulnar lengthening with an external fixator and/or excision of the distal ulnar osteochondroma. The lengthening began 10 days after the surgery with distraction at a rate of 0.75 mm/day. The anticipated lengthening should meet the two following criteria. Proximally, the radial head should pull the trochlear notch of the ulna, while distally, the positive ulnar variance should be obtained by overlengthening at 5–10 mm. The obtained lengthening was measured and recorded on a lateral film on the last day of lengthening.

The parametric Kolmogorov-Smirnov test was used to check for normal distribution of the data. A one-way analysis of variance was used to compare the continuous variables. Post hoc tests were used to compare the radiologic values between pre-op and different follow-up stages; Levene’s test was used to compare the variance between patients with Masada type I and type IIb deformity. A *p* value < 0.05 was considered statistically significant. All statistical analyses were conducted using the Statistical Package for the Social Sciences (SPSS Inc., Chicago, IL) version 17.0.

## Results

This study included fifteen patients (17 forearms) with a mean age of 9.4 ± 2.3 years at the index surgery. The mean follow-up period for all patients was 4.2 ± 2.4 years. The main complaints include cubitus varus in all patients, protrusion that results from the dislocated radial head at the elbow in all type IIb patients, and large bump (osteochondroma) at the distal ulna (4 in type I deformity and 6 in type IIb). The functional impairment was only noted by 4 patients’ parents; all of them were Masada type IIb patients. All patients were free of pain preoperatively and at the last follow-up. The mean ulnar lengthening was 4.2 ± 1.2 cm. Ten forearms were excised of osteochondromas concurrently at the distal ulna; two forearms have a history of osteochondromas removal before. There were 9 patients (10 forearms) who were Masada type I, with a mean age of 8.2 ± 1.5 years, and 6 patients (7 forearms) were type IIb, with a mean age of 11.0 ± 2.2 years. The preoperative data was compared between patients with Masada type I and Masada type IIb. The ulnar was significantly shorter for patients with Masada type IIb deformity (*p* = 0.021), demonstrating less pronation and flexion than those with type I deformity (*p* = 0.015 and 0001, respectively) (Table 1).

**Table 1:** Characteristics of 17 forearms before operation (mean ± SD)

	Patient with Masada type I deformity	Patient with Masada type IIb	*p* value
Age (mean years ± SD)	8.2 ± 1.5	11.0 ± 2.2	0.006
Length of follow-up	3.6 ± 1.4	5.0 ± 3.4	0.333
RAA (°)	34 ± 4	42 ± 11	0.106
US (mm)	− 24 ± 7	− 40 ± 14	0.021
CA (°)	− 9 ± 5	− 3 ± 9	0.077
Pronation (°)	56 ± 16	33 ± 19	0.015
Supination (°)	93 ± 9	85 ± 22	0.400
Arc of forearm rotation (°)	149 ± 20	117 ± 33	0.028
Flexion (°)	141 ± 6	127 ± 6	0.001
Lack of extension (°)	1 ± 3	3 ± 6	0.273
Arc of elbow motion (°)	140 ± 6	124 ± 5	< 0.001

The mean values of the radiographic and clinical assessment parameters are integers

*RAA* radial articular angle, *US* ulnar shortening, *CA* carrying angle

The preoperative, postoperative, and last follow-up RAA and US values are shown in Table 2. Initially, the mean RAA was improved significantly after external fixator removal (*p* = 0.005); relapse occurred at the last follow-up. The mean change in US was − 30 ± 13 mm preoperatively and was reduced significantly to 8 ± 5 mm overlength of the ulna after fixator removal (*p* < 0.001). There was some regression with a mean − 8 ± 9 mm of ulnar shortening at the last follow-up examination, but overall, there was a significant improvement compared to pre-op (*p* < 0.001).

**Table 2:** Radiographic parameters assessment at different follow-up stages (mean ± SD)

	Preoperative	Postoperative	Last follow-up	*P*_1_ value	*P*_2_ value
RAA (°)	37 ± 8	30 ± 7	34 ± 7	0.005	0.255
US (mm)	− 30 ± 13	8 ± 5	− 8 ± 9	< 0.001	< 0.001

*RAA* radial articular angle, *US* ulnar shortening, *P*_*1*_ data between the pre-op and post-op, *P*_*2*_ data between the pre-op and the last follow-up

The carrying angle improved significantly from − 6 ± 7° preoperatively to 7 ± 11° at the last follow-up (*p* < 0.001). The forearm pronation also improved significantly, from 47 ± 20° preoperatively to 73 ± 16° at the last follow-up (*p* < 0.001). The mean supination was 90 ± 16° preoperatively, which decreased to 84 ± 11° at the last follow-up. Despite this slight deterioration (*p* = 0.155), the total arc of the forearm rotation improvement was still significant. The mean flexion was 135 ± 9° preoperatively, increased to 140 ± 9° (*p* = 0.013) at the last follow-up. The mean extension limitation of the elbow was measured as 2 ± 4° preoperatively and 3 ± 5° at the last follow-up, and the arc of the elbow motion increased from 134 ± 9° preoperatively to 137 ± 10° at the last follow-up, both of them remained unchanged (*p* = 0.351 and 0.055, respectively).

The functional results for patients with Masada type I and IIb are shown in Tables 3 and 4, respectively. Besides the pronation and arc of forearm rotation, elbow flexion and the arc of elbow motion improved significantly in patients with type IIb deformity (*p* = 0.027 and 0.023, respectively).

**Table 3:** Range of movement and carrying angle in patients with Masada type I based on follow-up stage (mean ± SD)

	Preoperative	Last follow-up	*p* value
CA (°)	− 9 ± 5	6 ± 12	0.004
Pronation (°)	56 ± 16	79 ± 16	0.003
Supination (°)	93 ± 9	91 ± 6	0.599
Arc of forearm rotation (°)	149 ± 20	170 ± 19	0.005
Flexion (°)	141 ± 6	143 ± 8	0.223
Lack of extension (°)	1 ± 3	2 ± 5	0.279
Arc of elbow motion (°)	140 ± 6	141 ± 9	0.798

*CA* carrying angle

**Table 4:** Range of movement and carrying angle in patients with Masada type II based on follow-up stage (mean ± SD)

	Preoperative	Last follow-up	*p* value
CA (°)	− 3 ± 9	11 ± 7	0.005
Pronation (°)	33 ± 19	65 ± 12	0.002
Supination (°)	85 ± 22	75 ± 10	0.193
Arc of forearm rotation (°)	117 ± 33	140 ± 17	0.032
Flexion (°)	127 ± 6	137 ± 10	0.027
Lack of extension (°)	3 ± 6	4 ± 6.0	0.689
Arc of elbow motion (°)	124 ± 5	133 ± 10	0.023

*CA* carrying angle

In patients with Masada type I deformity, no radial head dislocation was reported at the last follow-up except for one who suffered a callus fracture after external fixator removal and progressed to type IIb deformity during the follow-up. In patients with type IIb deformity, a similar fracture also occurred in one patient, and no patient had a concentric reduction of the radiocapitellar joint after external frame removal (Fig. 1).

## Discussion

The osteochondroma-induced growth disturbance of the distal ulnar physis usually leads to unbalanced growth between the radius and ulna, resulting in the functional limitation of the forearm and elbow, as well as an unappealing cosmetic appearance of the arm. Ulnar lengthening by distraction osteogenesis with or without an associated procedure has been the most prevalent procedure in the treatment of Masada type I/II deformity [9]. However, the necessity of operation for such deformities is still controversial, and appropriate treatment is challenging, especially for children. The core of these problems is the concern of recurrence after surgery in skeletally immature patients [2, 10, 11] and less restriction of daily life activities in adult patients. Stanton and Hansen [12] and Arms et al. [13] suggested less aggressive corrective surgery for forearm deformities because skeletally mature patients have a good tolerance to minimal functional impairment and appearance deformity. Noonan et al. found that only 13% of untreated adult patients, often with varying degrees of functional deficit, had substantial pain or limitations related to their job performance [14], and patients were found adaptable and with excellent QuickDASH questionnaire responses in the previous study [10]. Furthermore, some long-term follow-up studies reported a high risk of recurrence of early intervention in skeletally immature patients [15, 16].

The previous studies were focused on the function impairment of the forearm deformity. However, unlike adult patients, self-assessed functional impairment and patient satisfaction can’t be evaluated in children. The indications for surgery in the present study were cosmetic forearm deformity, cubitus varus, and/or protrusion of the radial head at the elbow and the large bump (osteochondroma) at the distal ulna and were the initial problems noted by their parents, while functional limitations were observed through examination by a doctor rather than by the parents.

We advocated early ulnar lengthening in children for such deformities for several reasons as follows. Our data confirm that forearm deformity progresses with age, and functional impairment in Masada type IIb was severer (Table 1). In agreement with Clement and Porter [1], the present study considers that the proximal migrant radial head restricts forearm rotation and elbow flexion. In this series, even without a complete reduction of the radial head, the cosmetic appearance and function improved significantly after ulnar lengthening due to the radial head position improvement. At the last follow-up, the range of motion of both the elbow and forearm increased significantly. Furthermore, reducing a dislocated radial head might be more difficult than preventing a radial head subluxation. In patients with Masada type I deformity, gradual ulnar lengthening with or without the osteochondromas removal can maintain a normal radiocapitellar joint and less functional impairment. In this study, good congruency of the radiocapitellar joint was observed in all patients with type I deformity except one. By contrast, no concentric reduction of the radiocapitellar joint was obtained in patients with type IIb deformity. The adaptive lateral angulation of the proximal radius (Fig. 1) and other pathological changes of the soft structure of the elbow due to the long-standing dislocation of the radial head can explain this phenomenon. Thus, simple ulnar lengthening and other ligament reconstruction surgeries are rarely successful in such conditions [15]. Similar results were reported by Hsu et al., who treated 14 patients by ulnar lengthening and suggested ulnar lengthening alone in treating patients younger than 10 years, while additional radial corrective osteotomy to correct the radial bowing deformity for older than 10 years patients [17]. Meanwhile, Iba and colleagues reported good results by using simple axis correction and ulnar osteogenesis distraction without corrective osteotomy of the radius to treat radial head dislocation within 1 year in HMO patients [18]. In another study, the short interval between the occurrence of radial head dislocation and surgery was considered the key to a successful procedure [19]. The above studies showed that the earlier the intervention, the simple the procedure and the better the outcomes.

No patient provided the exact time of radial head dislocation in this study. However, older age and severer ulnar shortening indicated the long history of the dislocation, resulting in a more prolonged distraction stage and higher complications rate. In addition, we found that in patients with severe dislocation, the pulling force of the interosseous membrane is limited and could no longer pull the radius moving distally after a large amount of lengthening; continuous traction only resulted in a positive ulnar variance without any displacement of the radial head (Fig. 1). Our unsatisfied results of radial head reduction support early ulnar lengthening for patients with Masada type IIb deformity.

**Fig. 2:**
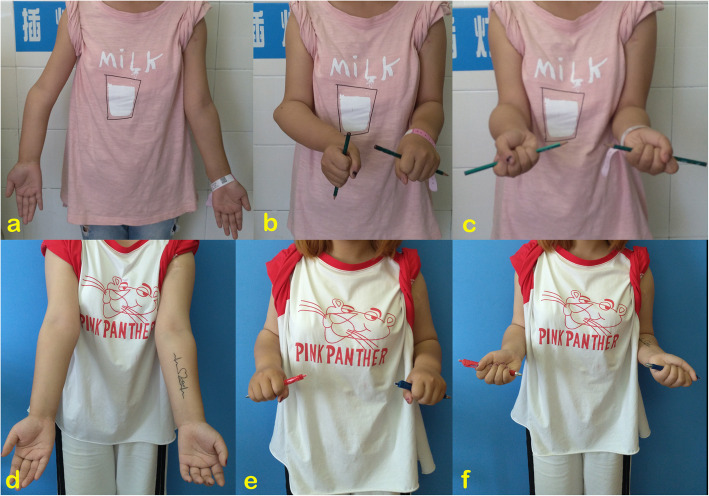
**a–c** A 12-year-old girl with type IIb deformity. Her parents complained about an obvious cubitus varus and protrusion of the radial head at the elbow but did not notice functional limitation of the forearm. **d–f** Both appearance and rotation improved at 2.5 years follow-up

We consider that the recurrence of ulnar shortening is inevitable in skeletally immature patients. Some studies reported satisfactory results after ulnar lengthening, but the mean follow-up times of their patients were short (< 2.5 years) [3, 4], or the age at intervention was older than 10 years [5]. In a study with longer-term follow-up, Akita et al. reported recurrence in children who underwent surgery too early [15], and similar results were reported by Litzelmann et al. [10]. Therefore, Abe et al. proposed postponing ulnar lengthening until after 10 years old to avoid the recurrence of deformities [20]. Ham et al. even suggested delaying the lengthening until after 13–15 years old [16]. We believe that long-term follow-up leads to more convincing results; however, our results of a mean follow-up of 4.2 years also provided valuable reference data. Although not all patients were followed-up to the end of the growth spurt, the mean age at the last follow-up was 13.5 years approximately to adolescent. In such a period, we can evaluate the effect of ulnar lengthening and observe the recurrence of the deformity. Although a partial recurrence of ulnar shortening was observed, the ulnar shortening and forearm pronation improved significantly by ulnar lengthening, and the cosmetic appearance improved significantly with the change of carrying angle (Fig. 2).

As described previously in the literature, mild ulnar shortening was acceptable in patients [2, 21], repeated lengthening is not recommended unless a progressive radial head dislocation is detected. We performed 5–10-mm overlengthening of the ulna in this study, but the limited overlengthening of the ulna seems not useful in the prevention of recurrence in skeletally immature patients. As the natural course of the deformities is unpredictable, the exact length discrepancy between the radial and ulna at skeletal maturity is difficult to predict. Furthermore, the forearm comprised two bones, the ulna and the radius, which articulate with each other at the proximal and distal radioulnar joints. Thus, unlike one bone lengthening, the ulna cannot be lengthened to the anticipated length at one stage without considering the balance between the radius and ulna. In addition, some authors proposed that ulnar overlengthening could result in ulnocarpal impaction and should not be recommended even in patients with a higher risk for recurrence [22, 23]. In contrast, Hill et al. mentioned that overcorrection was partially successful at preventing subluxation or dislocation of the radial head [24]. We share the same experience; we have no patient complained about the pain of the wrist joint, and even by overlengthening, slight ulnar shortening still exists at the last follow-up. We consider minimal overlengthening of the ulnar could partially compensate for the growth imbalance between the radius and the ulna (Fig. 1) and could thus delay the second lengthening of the ulna.

**Fig. 3:**
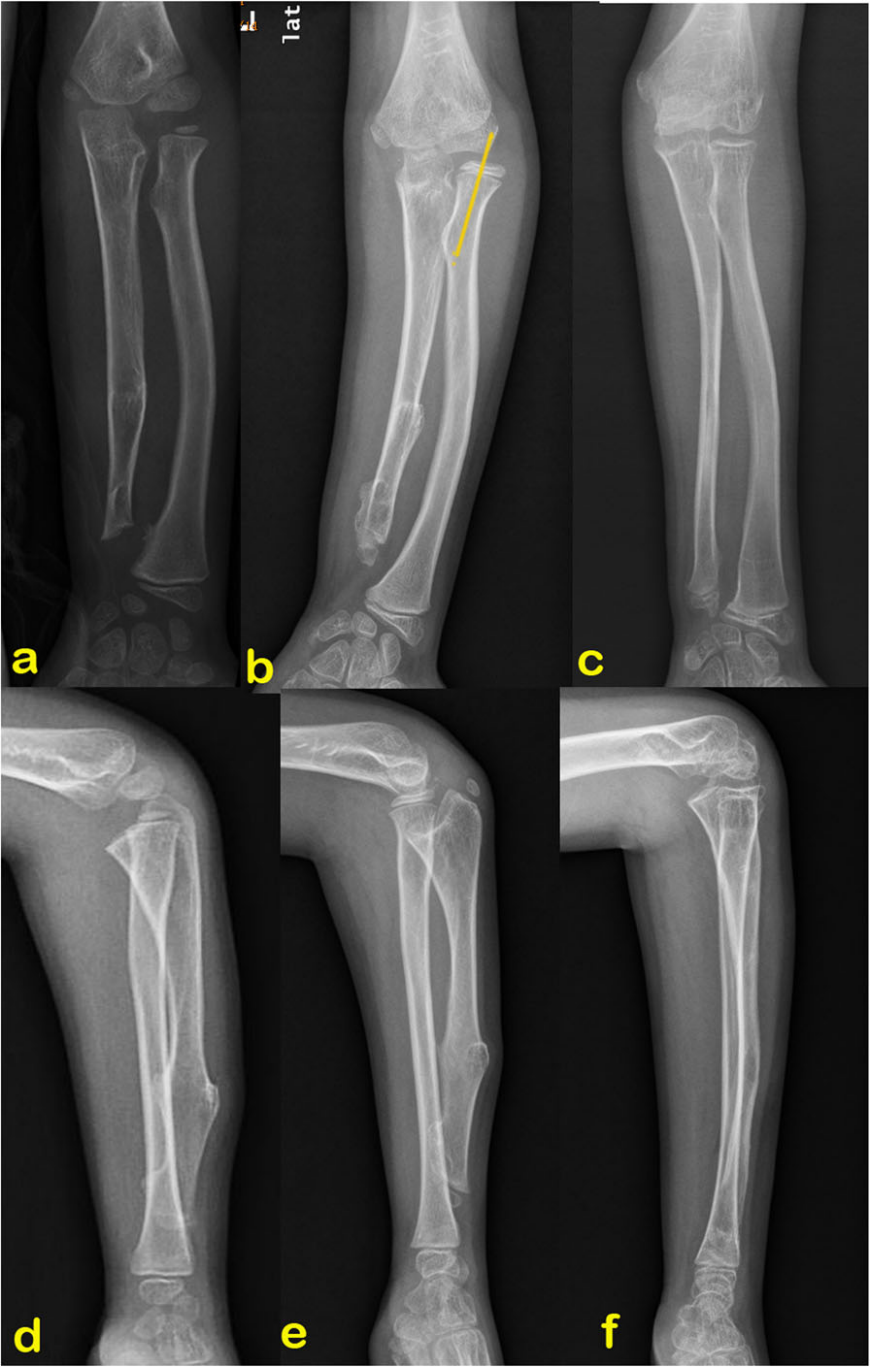
**a**, **b** Forearm deformity progressed with age, radial head proximal migrant and the radial neck axis lateral deviation were found 3 years later after the first clinical assessment, and ulnar gradual lengthening with osteochondroma resection was performed at a 10-year-old. **d**, **e** Lateral radiograph of the same patient. **c**, **f** 2.1 years after the external fixator was removed, both the proximal radioulnar joint and the radiocapitella joint were well maintained

Unlike reported previously in the literature, the age that ulna lengthening should be performed is not fixed and is dependent on the severity of the deformities in the present study. Matsubara et al. proposed that the recurrence of ulnar shortening might depend more on the extent of damage to the distal ulnar physis [22]. We recommend an individualized treatment plan for Masada type I deformity. In our study, surgery is not usually arranged at the patient’s first clinical assessment, unless a tendency of dislocation of the radial head is observed (Fig. 3). For Masada type IIb deformity with new-onset radial head dislocation, the ulnar lengthening can be postponed no more than 1 year.

There are a few limitations of this study that warrant discussion. First, this is a retrospective study with a small number of patients. Second, we did not measure the ROM of the wrist joint and functional performance, which are important in evaluating the stability of the wrist. In addition, most patients in this study were skeletally immature at the last follow-up. Therefore, the recurrence risk could not be precisely evaluated. A longer follow-up duration and more detailed data of functional assessment are required to comprehensively assess the results of ulnar lengthening surgery. Furthermore, there is a lack of comparative studies in the literature with regard to functional outcomes for untreated, skeletally immature patients with HMO. Future randomized control studies are needed to better understand these issues. As reported by previous work [8, 15, 25], the pronation improvement may be partially due to the excision of the osteochondroma; these results warrant further control studies to specifically evaluate the effect of ulnar lengthening on forearm rotation. However, the results of a mean follow-up of 4.2 years showed satisfactory appearance improvement which was the main purpose of the surgery of the parents.

## Conclusion

Gradual ulnar lengthening significantly reduces cosmetic deformity and improves function in patients with Masada type I/IIb deformity. As only one patient with type I deformity progressed to type IIb at the last follow-up, no type IIb forearm obtained concentric reduction. Our results supported early ulnar lengthening for patients with a progressive radial head dislocation to maintain the stability of the radiocapitellar, even there may be a high risk for recurrence and need for repeated lengthening. For Masada type IIb deformity, ulnar gradual lengthening improved the function and cosmetic appearance rather than reducing the radial head.

## Data Availability

The datasets generated and/or analyzed during the current study are not publicly available because they contain patients’ personal information but are available from the corresponding author on reasonable request.

## Abbreviations

HMO: Hereditary multiple osteochondromas
CA: Carrying angle
ROM: Range of motion
RAA: Radial articular angle
US: Ulnar shortening
DASH: Disabilities of the Arm, Shoulder and Hand Score
